# Shedding Light on Fish Otolith Biomineralization Using a Bioenergetic Approach

**DOI:** 10.1371/journal.pone.0027055

**Published:** 2011-11-14

**Authors:** Ronan Fablet, Laure Pecquerie, Hélène de Pontual, Hans Høie, Richard Millner, Henrik Mosegaard, Sebastiaan A. L. M. Kooijman

**Affiliations:** 1 Institut TELECOM, TELECOM Bretagne, Lab-STICC, Brest, France; 2 Department of Ecology, Evolution and Marine Biology, University of California Santa Barbara, Santa Barbara, California, United States of America; 3 Ifremer, Departement of Fisheries Sciences and Technology, Plouzane, France; 4 Department of Biology, University of Bergen, Bergen, Norway; 5 Institute of Marine Research, Bergen, Norway; 6 CEFAS, Lowestoft, Suffolk, United Kingdom; 7 National Institute of Aquatic Resources, Technical University of Denmark, Charlottenlund, Denmark; 8 Department of Theoretical Biology, Vrije Universiteit, Amsterdam, The Netherlands; California Academy of Sciences, United States of America

## Abstract

Otoliths are biocalcified bodies connected to the sensory system in the inner ears of fish. Their layered, biorhythm-following formation provides individual records of the age, the individual history and the natural environment of extinct and living fish species. Such data are critical for ecosystem and fisheries monitoring. They however often lack validation and the poor understanding of biomineralization mechanisms has led to striking examples of misinterpretations and subsequent erroneous conclusions in fish ecology and fisheries management. Here we develop and validate a numerical model of otolith biomineralization. Based on a general bioenergetic theory, it disentangles the complex interplay between metabolic and temperature effects on biomineralization. This model resolves controversial issues and explains poorly understood observations of otolith formation. It represents a unique simulation tool to improve otolith interpretation and applications, and, beyond, to address the effects of both climate change and ocean acidification on other biomineralizing organisms such as corals and bivalves.

## Introduction

**Figure 1:**
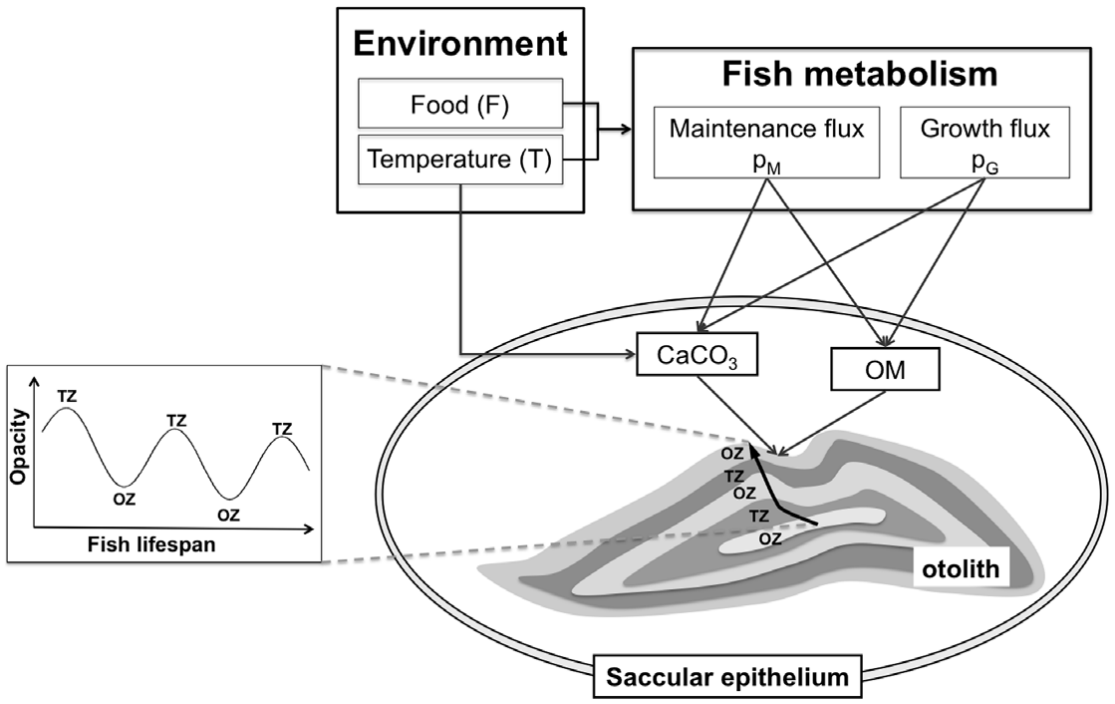
Model for otolith biomineralization. Otolith formation corresponds to an accretion of successive layers of calcium carbonate (CaCO_3_) embedded in an organic matrix (OM) which precursors are synthesized by the saccular epithelium. At a yearly scale seasonal environmental and physiological variations induce opacity changes with an alternated deposition of translucent (TZ) and opaque (OZ) zones appearing respectively as dark and bright zones under reflected light. We here state the otolith as a metabolic product as defined by the Dynamic Energy Budget (DEB) theory for metabolic organization [18]; Otolith formation is driven by fish growth (p_G_) and maintenance (p_M_) metabolic fluxes which depend on the individual state and the temperature and feeding conditions the fish experiences. We also account for the temperature-dependent dynamics of CaCO_3_ precipitation [15].

Otoliths, biomineralized aragonite bodies in the fish inner ear, have long been recognized as key biological archives. Many species deposit seasonally alternating opaque and translucent zones (Fig. 1) that provide proxies of age critical in fish population dynamics [1]. By providing dated morphological, structural and chemical signatures, otoliths are also keys for past and present environment reconstructions [2] and life trait characterization [3], [4], [5], [6]. Such data are critical for marine ecosystem and fisheries monitoring. Due to the poor understanding of biomineralization mechanisms, otolith proxies however often lack validation and are open to subjective interpretations [1], [7]. Inaccurate otolith-based age estimation of orange roughy off New Zealand [8] and walleye pollock in the Bering Sea [9] are among the most striking examples of misinterpretations that have contributed to the overexploitation of fish populations.

Both metabolism and temperature are known to play key roles in otolith biomineralization [1], [10], [11]. As highlighted by meta-analyses [1], [12], disentangling these two factors is however challenging. In temperate waters, the formation of translucent zones is generally considered to occur during winter whereas opaque zones would be formed during rapid growth periods in spring and summer (Fig. 1). However, this statement is often not valid. Opposite patterns have been reported as well as additional non-periodical zones that may lead to erroneous age and growth estimations [13]. Neither experimental studies monitoring temperature and feeding conditions [10], [11] nor proposed otolith biomineralization models [14], [15], [16] have been able to explain the complex interplay between fish metabolism and temperature on otolith formation. In particular, consideration of mineral factors alone [15] has been challenged by recent characterizations of the role of organic compounds in otolith biomineralization [17].

We here propose a bioenergetic model of otolith biomineralization in the framework of the Dynamic Energy Budget (DEB) theory [18] (Fig. 1). This general theory for metabolic organization describes how an organism assimilates and utilizes energy throughout its life cycle. The key feature here is the application of the concept of metabolic product, as defined by DEB theory [18]. The mineral and organic fractions of the otolith are regarded as individual metabolic products involving contributions from somatic growth (p_G_) and maintenance (p_M_) DEB energy fluxes (Fig. 1), and otolith opacity variations result from variations in the ratio between these two fractions [19]. Given that in vitro aragonite precipitation is temperature-dependent [15], temperature variations also directly act on the dynamics of the mineral fraction (Fig. 1). Mathematically, given the parameterization of metabolic fluxes p_G_ and p_M_ defined by DEB theory [18], otolith growth (Eq. 1) and opacity (Eq. 3) can be regarded as functions of the state of the individual (reserves and length) and of its environment (temperature and food density). The 1D simulation of otolith formation may be transformed into a 2D transverse section image of a growing otolith using calibrated shape deformation algorithms for otolith images [20].

## Results and Discussion

**Figure 2:**
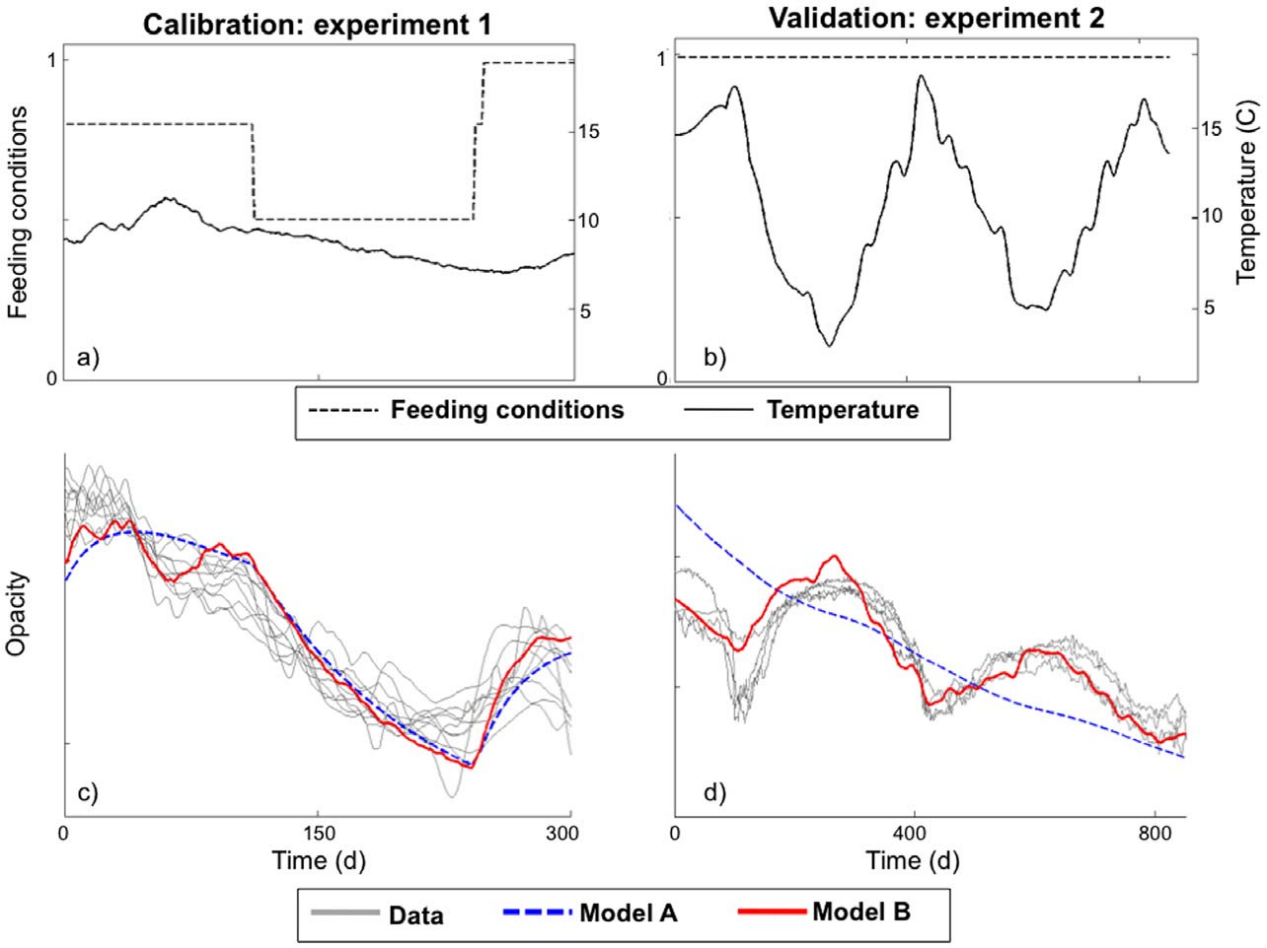
Model calibration and validation on cod otoliths using two experimental datasets: 1) reduced feeding conditions (day 110 to day 220) with seasonal temperature variations (left column), and 2) constant feeding with seasonal temperature cycles over a two-and-a-half-year period (right column). We report temperature and feeding conditions (a, b) and the comparison between model simulations and opacity data (c, d). We display opacity data (Data, gray) and model simulations without the temperature effect on calcium carbonate precipitation (Model A, blue) and with this temperature effect (Model B, red).

Model calibration and validation were carried out from two experimental cod otolith datasets. The calibration relied on a 300-day experiment on juvenile cod which experienced a shift to lower feeding conditions and varying temperatures (Fig. 2a; Fig. S1 & S2 and Table S1 & S2). The validation involved a 800-day experiment on juvenile cod which experienced seasonal temperature variations and constant feeding (Fig. 2b; Fig. S3 & S4). Metabolic effects alone induced most of the opacity variations in the first experiment but could not explain seasonal opacity signals in the second experiment (Fig. 2; Fig. S2 & S4). Temperature factor *c_C_(T)* was negatively correlated to opacity in the first experiment (Fig. 2, left column) and could not account for the overall decreasing opacity trend in the second experiment (Fig. S4). Only the interplay between the metabolic and temperature factors led to a reliable prediction (R^2^>0.9, p<0.001 in both cases). These results also outlined the different dynamics of feeding and temperature effects. Whereas temperature acted immediately through the regulation factor *c_C_(T)*, food-induced effects were typically smoothed out, the reserves of the individual acting as a buffer.

**Figure 3:**
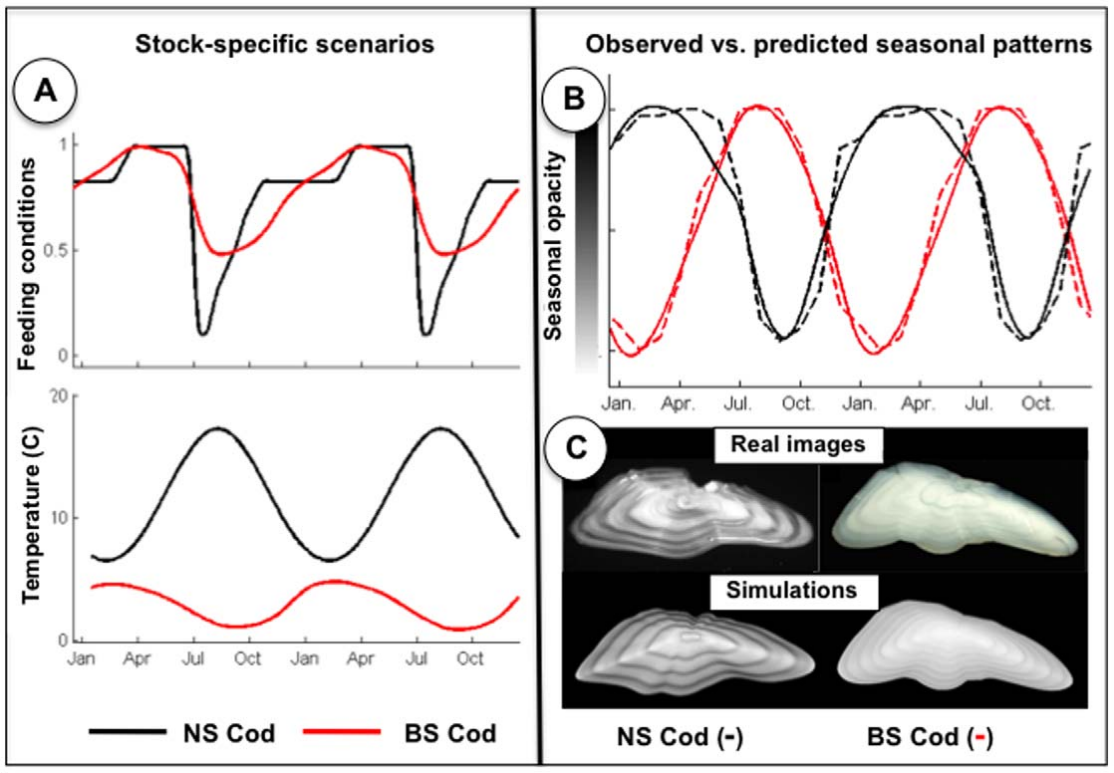
Resolving the non-synchronous seasonality of opacity patterns of Barents Sea (BS) and southern North Sea (NS) cod otoliths: Feeding and temperature conditions (panel A) that explain otolith opacity patterns observed for southern North Sea (NS, black) and Barents Sea (BS, red) cod (panels B and C). Observed seasonal patterns (dashed lines), given as the relative proportions of opaque edges in the monthly sampled otolith sets [21], are compared to normalized simulated opacity patterns (solid lines). Model simulations reproduce both the opposite seasonal opacity patterns (panel B) and the remarkable differences in the contrast of the otolith images of the two populations (panel C). The Supp. Mat. details the stock-specific scenarios (Text S1 & Fig. S5) and animated model simulations are provide as an electronic appendix (Video S1).

The proposed model opens up new prospects for the understanding of differences in otolith patterns of a given species within different ecosystems (Fig. 3). As an illustration, we considered two cod populations respectively in the Barents Sea (BS) and in the southern North Sea (NS) (Fig. 3). Their otoliths depict antiphasic seasonal opacity patterns (Fig. 3B). BS cod follows the general pattern with a winter translucent zone and an summer opaque zone, while NS cod forms an opaque zone in spring and a translucent one in late summer [21]. In addition, NS cod otolith images are much more contrasted than BS cod ones (Fig. 3C). By forcing the calibrated model with population-specific feeding and temperature scenarios stated from data available in the literature (Fig. S5, and Video S1), we explained these two population-specific characteristics. The smaller variations in both feeding and temperature conditions experienced by the BS cod result in otolith images with a lower contrast well redrawn by the model (Fig. 3C). Observed seasonal patterns (dashed lines, Fig. 3B), given as the relative proportions of opaque edges in monthly sampled otolith sets [21], were compared to normalized versions of the simulated opacity patterns (solid lines, Fig. 3B; Fig. S6). The model convincingly reproduced the seasonal patterns (R^2^>0.96 p<0.001). Neither of the two populations conforms to the generally assumed interpretation, i.e. slow-growth winter translucent zones and fast-growth summer opaque ones [1]. BS cod forms a late winter translucent zone which is induced by migration to warmer waters rather than slow-growth conditions (Fig. S8). The opposite pattern of the NS cod results from the late summer formation of a translucent zone due to low feeding activity with simultaneous high temperatures (Fig. S7). Besides, we showed that similar seasonal opacity patterns for different populations, here Barents sea cod and Norwegian coast cod populations, might not necessarily refer to similar feeding and temperature conditions but might also be observed with different population-specific scenarios (Fig. S9). These results highlight the complex interplay between temperature and feeding conditions each of which may individually have a positive or a negative effect on otolith growth and opacity. These interactions as well as the above-mentioned differences in their relative response dynamics explain why empirical studies have reached contradictory conclusions on the regulation of the formation of otolith structures among species and stocks [12].

Improving the reliability of otolith-based individual and population data is critical to population dynamics and ecology. In this respect, our model provides a conceptual basis to interpret well-known but poorly understood otolith characteristics:

- The coupling between otolith growth and fish somatic growth during high feeding periods [10], [22] results from the large contribution of the somatic growth flux (α_C_p_G_>>β_C_p_G_, Eq. 1). In contrast, low feeding periods [10], [22] lead to a decoupling due to the weaker but significant contribution of the maintenance flux in otolith growth (α_C_p_G_∼0 and β_C_p_G_>0, Eq. 1);
- The correlation between otolith growth and fish respiration [23] follows naturally as CO_2_ production is also modelled as a weighted sum of metabolic processes in a DEB context [18];
- The differences in the relative contributions from the somatic and maintenance fluxes (Eq. 3) result in metabolism-induced opacity changes; improved feeding conditions lead to a more opaque accretion [21]. They also explain the lifespan decrease of opacity as the growth flux decreases as the individual gets closer to its asymptotic size [19];
- The greater otolith accretion at higher temperatures [11], [24] is a direct outcome of the temperature-dependent dynamics of the precipitation of aragonite (Eq. 1). This mechanism also accounts for the formation of a more opaque otolith zone when the fish experiences colder temperatures [11], [24].

Beyond these new mechanistic interpretations, scenario-based model simulations are of primary interest to interpret and predict otolith characteristics in response to environmental changes (e.g. climate). For instance, they provide new means for the discrimination of seasonal vs. non-seasonal otolith structures, a crucial issue for the improvement of the accuracy of individual age data [1]. Direct model inversion also presents a great potential for the reconstruction of individual life traits from otolith patterns. For instance, given temperature records obtained from data storage tags or estimated from the oxygen isotopic ratios of the otolith, modeled otolith accretion and opacity may be fitted to the recorded macrostructures of real otoliths by tuning individual feeding dynamics and growth. To our knowledge, the acquisition of feeding dynamics at the individual scale remains a challenge in non-monitored environments, but it is particularly important for the understanding and prediction of food web dynamics. The analysis of otolith chemical composition could also benefit from the proposed framework. Both element and isotopic signatures provide invaluable information on fish migration and population connectivity [4], [6]. However, they often depict complex interactions between endogenous and environmental factors [7] that may be deciphered by extensions of our approach.

The biomineralization of other structures such as coral skeletons and bivalve shells also lacks a comprehensive understanding. The proposed framework provides a generic basis for modeling their formation. The biomineralization mechanisms we considered, a metabolism-driven control parameterized by somatic growth (assumption A1) and maintenance energy fluxes and a temperature-specific effect on precipitation dynamics and (assumption A2) are generic and their implementation exploits a theory for metabolic organization already applied to fish, bivalves and corals [25], [26]. DEB-based biomineralization models could then provide simulation tools to addres the effects of climate change on a large variety of calcifying organisms [27]. Furthermore, by providing a framework where pH conditions could impact *i)* metabolic processes and *ii)* CaCO_3_ precipitation directly and indirectly via their impact on metabolic processes, we strongly believe that these models represent a promising starting point to investigate the consequences of ocean acidification on biocalcifying organisms [28].

## Methods

### A generic model of otolith formation

The biomineralization of otoliths is primarily controlled by organic compounds in the endolymph [17]. These organic compounds being synthesized by specialized cells of the saccular epithelium, we here relate otolith formation to fish bioenergetics in the framework of the DEB theory [18]. Our model relies on two basic assumptions:

- *A1-Both the aragonite fraction and the organic matrix of an otolith are metabolic products.* In DEB theory, such compounds are formed during metabolic processes but do not require maintenance and are not used to fuel other metabolic processes [18]. This applies to fish otoliths as they are inert biomineralized structures whose formation is primarily controlled by physiological factors [17];
- *A2-The precipitation of the mineral fraction of the otolith is temperature-dependent.* This assumption is supported by in-vitro analysis of aragonite precipitation [15].

From (A1), the dynamics of the volumes of the mineral and organic fractions of the otolith, respectively *V_C_* (µm^3^) and *V_P_* (µm^3^), are derived as functions of the somatic growth flux (*p_G_*, J.d^−1^) and the maintenance flux (*p_M_*, J.d^−1^) of an individual fish:
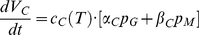
(1)

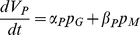
(2)where α_C_, β_C_, α_P_, β_P_ (µm^3^.J^−1^) are model parameters. The regulation factor *c_C_(T)*, stated as an Arrhenius law (Text S1, Section 1), accounts for the temperature effect on mineral precipitation dynamics (A2). As defined by DEB theory [18], the growth and maintenance fluxes (*p_G_* and *p_M_*) are functions of the state of the individual (reserves and length) and of its environment (temperature and food density) (Text S1, Section 1). Given that the organic fraction accounts for less than 5% of the otolith volume [17], we neglect its contribution and the otolith volume is predicted by the volume of the mineral fraction.

Otolith opacity *O* relates to variations in the ratio between the volumes ΔV_P_ and ΔV_C_ of the organic and mineral fractions of the newly precipitated material [19]:
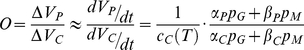
(3)The temporal simulation of otolith formation is transformed into a 2D transverse section image of an otolith using calibrated shape deformation algorithms [20]. This allows comparing simulated otolith images to real ones. We let the reader refer to the Supp. Mat. for further details on the modeling assumptions (Text S1, Section 1) and model parameters (Table S1 & S2).

### Model validation and calibration

We used otolith data from two different cod rearing experiments for model calibration and validation. In Experiment 1, one-year-old fish ranging from 30 to 35 cm were reared under seasonal temperature variations in high feeding conditions for 100 days, lower feeding conditions for the subsequent 120 days and ad libitum conditions for the last 80 days [10]. In Experiment 2, fish were 7 months old at the start of the experiment. They were fed ad libitum for 22 months and experienced seasonal temperature conditions [29]. In both cases, calibrated otolith data (i.e., time-referenced otolith growth and opacity data) were available along with the fish growth data.

The otolith data from Experiment 1 along with published data [30] were used to calibrate the DEB otolith model and the dataset from Experiment 2 was used as a validation dataset. The Supp. Mat. (Text S1, Table S1 & S2) further details model calibration and validation and reports calibrated model parameters.

### Analysis of seasonal otolith patterns

We applied the calibrated cod otolith model to the analysis of the opposite seasonal opacity patterns of two cod populations, namely Barents Sea cod and Southern North Sea cod [21]. The definition of two population-specific feeding and temperature scenarios relied on data available in the literature (Fig. S5):

- For the NS cod population, the yearly temperature conditions are given by the dynamics of surface temperatures in the southern North Sea [21]. Following [31], mid-level and high-level feeding conditions were respectively assumed from December to February and between March and July while a low feeding behaviour corresponding to temperature highs was considered from August to October;
- For the BS cod population, the considered temperature conditions were issued from records of data storage tags [32] showing a long southward migration to warmer temperatures in winter. In accordance with this seasonal migration, we assumed that feeding conditions improved in the winter and spring with a peak in feeding conditions, corresponding to the seasonal feeding on capelin in March–April [33], followed by lower feeding conditions form August to November prior to the start of the southward migration in December.

For the two populations, we compared simulated otolith images to real ones as well as the observed and predicted seasonal opacity patterns. These observed seasonal opacity patterns from [21] were given as the percentage of opaque otolith edges for monthly sampled cod otolith sets. The seasonal patterns of the model simulations were issued as detrended and normalized version of the predicted opacitie series.

## Supporting Information

Figure S1 **Model simulations for a shift in feeding conditions (Exp. 1): first row, feeding conditions, temperature conditions (a–b); second row somatic and otolith distal radius (c–d).** Model simulations (red) are compared to otolith data (gray) for the known feeding and temperature conditions. The model parameters are given in Tables S1 and S2.
(TIF)

Figure S2 **Model simulations for a shift in feeding conditions (Exp. 1): otolith data (gray, thin solid lines) for the known feeding and temperature conditions are compared to the model simulations for two parameter settings: a model with no temperature-specific effect (i.e., parameter T_AC_ set to 0) (R^2^ = 0.93, p<0.001, blue dashed line) and the calibrated otolith model (Table S1 & S2) (R^2^ = 0.96, p<0.001, red, solid line).**
(TIF)

Figure S3 **Model simulation for constant feeding conditions and seasonal temperature cycles (Exp. 2): first row (from left to right), feeding and temperature conditions (a–b); second row, somatic growth and otolith distal radius (c–d).** The simulation of the calibrated model (red) is compared to individual data (gray).
(TIF)

Figure S4 **Simulation of opacity patterns for constant feeding conditions and seasonal temperature cycles (Exp. 2).** Real opacity data (gray, thin solid lines) are compared to three different simulations: a simulation of the calibrated model (Table S1 & S2) (red, solid line), a simulation with no temperature regulation (blue, dashed line) and a simulation where otolith opacity depends only on temperature (magenta, dashed-dotted line). The correlation coefficients with the real data were R^2^ = 0.90, R^2^ = 0.66 and R^2^ = 0.43, respectively (p<0.001 in all cases).
(TIF)

Figure S5 **Model simulations for Southern North Sea cod (NS, black) and Barents Sea cod (BS, red): food density series (a), temperature series (b), somatic growth patterns (c), and otolith opacity patterns (d).** The somatic growth data (panel c, dashed lines) were obtained from Bolle et al. (Jørgensen 1992) for the both populations.
(TIF)

Figure S6 **Seasonality of the timing of otolith zone formation for the simulated and real data for NS and BS cod: feeding conditions (a), temperature conditions (b), and seasonal opacity patterns (c).** BS cod are represented by red and NS cod by black. We compared the average proportions of translucent otolith edges for real otoliths taken from Høie et al. (Høie, Millner et al. 2009) (dashed lines) to identify simulated seasonal opacity patterns (solid lines).
(TIF)

Figure S7 **Seasonal otolith opacity patterns for NS cod with constant and non-constant feeding conditions: feeding conditions (a), temperature conditions (b), and seasonal opacity patterns (c).** We display two simulations: the one reported in Fig. S6 (solid lines, R^2^ = 0.96, p>0.001) and a scenario assuming a constant feeding with the temperature conditions used in Fig. S6 (dotted lines, R^2^ = 0.64, p>0.001). Simulated opacity patterns are compared to the otolith data (dashed, see Fig. S5).
(TIF)

Figure S8 **Seasonal otolith opacity patterns for BS cod with constant and non-constant feeding conditions: feeding conditions (a), temperature conditions (b), and seasonal opacity patterns (c).** We display two simulations: the one reported in Fig. S6 (solid lines, R^2^ = 0.96, p>0.001) and a scenario assuming a constant feeding with the temperature conditions used in Fig. S6 (dotted lines, R^2^ = 0.54, p>0.001). The simulated opacity patterns are compared to the real otolith data (dashed line, see Fig. S5).
(TIF)

Figure S9 **Seasonality of the timing of otolith zone formation for BS and Norwegian coastal (NC) cod: feeding conditions (a), temperature conditions (b) and seasonal opacity patterns (c).** BS cod are shown in red and NC cod in magenta. Both populations are known to display the same seasonal otolith opacity pattern. We compared the average proportions of translucent otolith edges for real otoliths taken from Høie et al. (Høie, Millner et al. 2009) (dashed lines) with simulated seasonal opacity patterns (solid lines).
(TIF)

Text S1 **This Supplementary Text provides further details and analysis regarding the key aspects of the proposed bioenergetic model of otolith biomineralization.**It is organized as a report and involves three main sections:
**1. A generic bioenergetic model of otolith biomineralization.** This section further details model assumptions and equations.
**2. Model calibration and validation.** This section details calibration and validation dataset and results, and report calibrated model parameters.
**3. Resolving the seasonal timing of the formation of opaque and translucent zones in fish otoliths of different cod populations.** This section details the analysis, from model simulations, of the non-synchronous and synchronous seasonal opacity otolith patterns of several cod populations, namely Barrents Sea, Southern North Sea and Norwegian coast cod populations.
(DOC)

Table S1 **Variables, parameter values and equations for individual growth and somatic maintenance in a standard DEB model.**
(TIFF)

Table S2 **Variables, parameter values and equations for otolith biomineralization.**
(TIFF)

Video S1 **Animated version of model simulations reported in** **Fig. 3** **.**
(MOV)
