# Supplementary figures and images for: Shedding Light on Fish Otolith Biomineralization Using a Bioenergetic Approach
Source: PLoS One. 2011 Nov 14;6(11):e27055. doi: 10.1371/journal.pone.0027055 (PMC3215717; doi:10.1371/journal.pone.0027055)

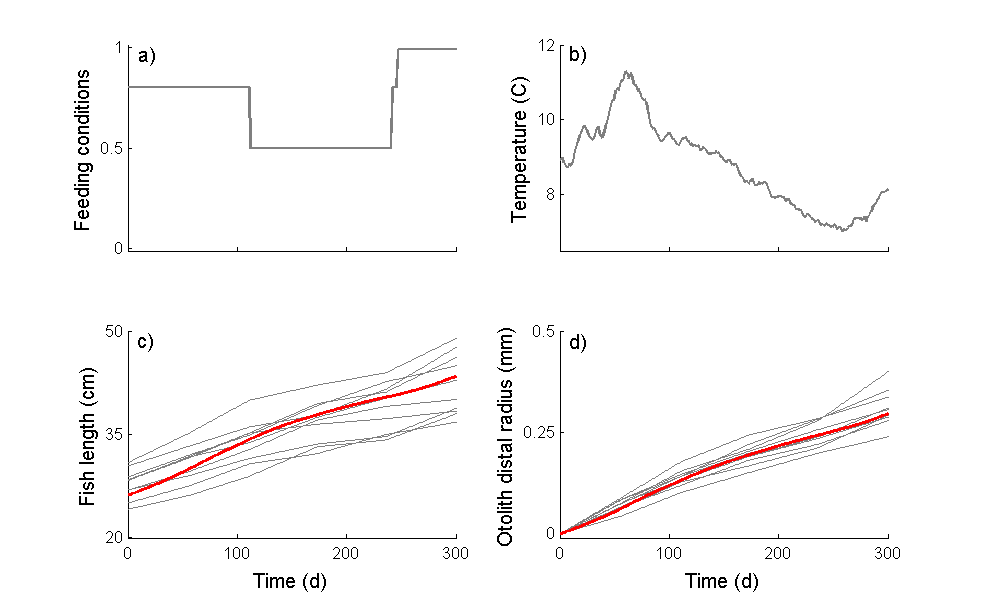

Supplement: Figure S1 — Model simulations for a shift in feeding conditions (Exp. 1): first row, feeding conditions, temperature conditions (a–b); second row somatic and otolith distal radius (c–d). Model simulations (red) are compared to otolith data (gray) for the known feeding and temperature conditions. The model parameters are given in Tables S1 and S2. (TIF) [file pone.0027055.s001.tif]

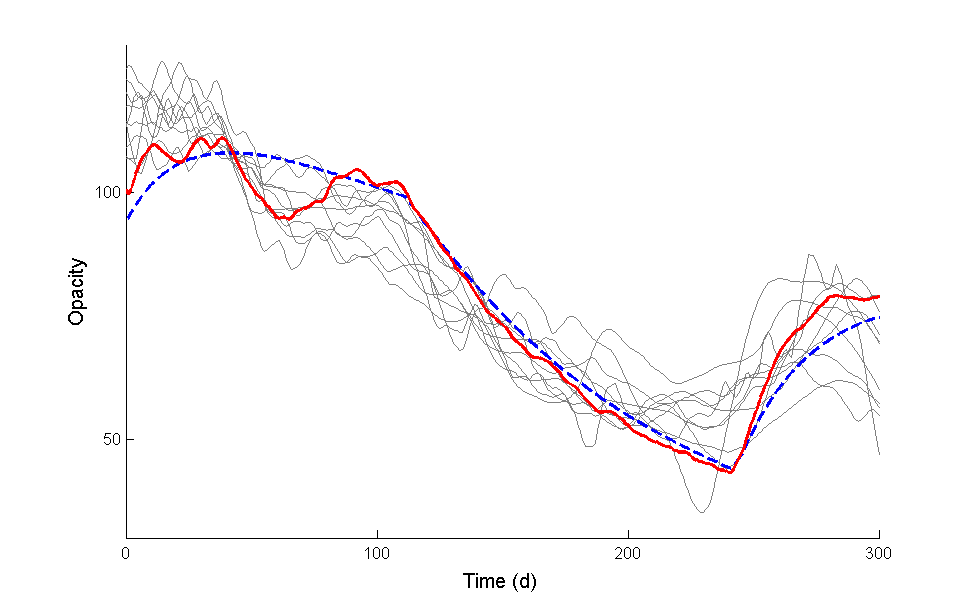

Supplement: Figure S2 — Model simulations for a shift in feeding conditions (Exp. 1): otolith data (gray, thin solid lines) for the known feeding and temperature conditions are compared to the model simulations for two parameter settings: a model with no temperature-specific effect (i.e., parameter TAC set to 0) (R2 = 0.93, p<0.001, blue dashed line) and the calibrated otolith model (Table S1 & S2) (R2 = 0.96, p<0.001, red, solid line). (TIF) [file pone.0027055.s002.tif]

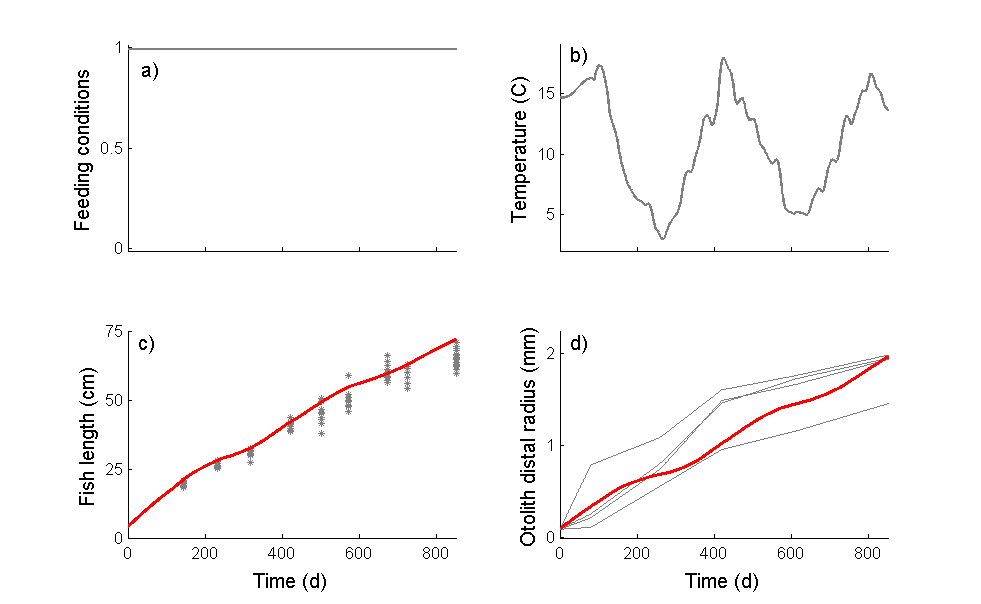

Supplement: Figure S3 — Model simulation for constant feeding conditions and seasonal temperature cycles (Exp. 2): first row (from left to right), feeding and temperature conditions (a–b); second row, somatic growth and otolith distal radius (c–d). The simulation of the calibrated model (red) is compared to individual data (gray). (TIF) [file pone.0027055.s003.tif]

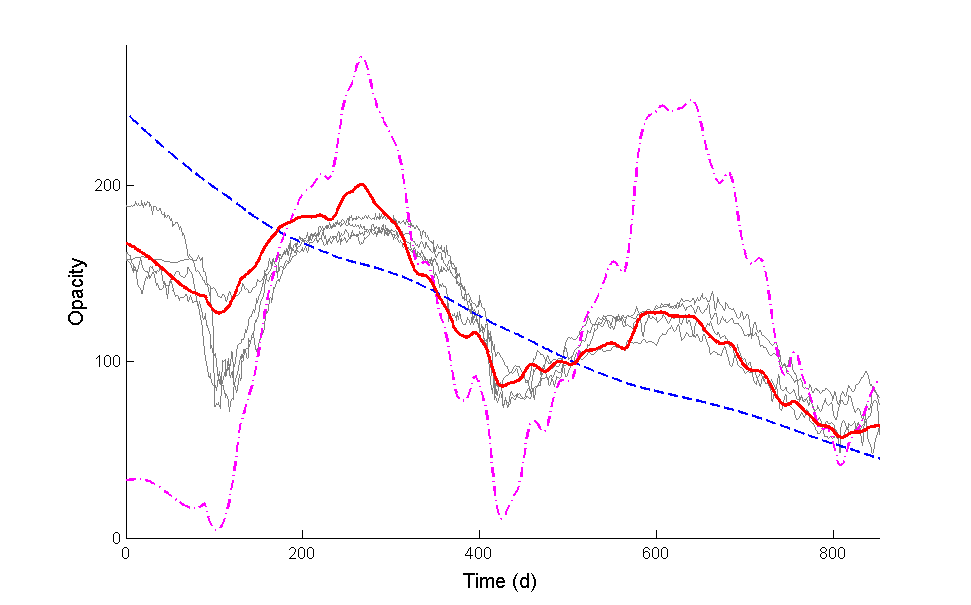

Supplement: Figure S4 — Simulation of opacity patterns for constant feeding conditions and seasonal temperature cycles (Exp. 2). Real opacity data (gray, thin solid lines) are compared to three different simulations: a simulation of the calibrated model (Table S1 & S2) (red, solid line), a simulation with no temperature regulation (blue, dashed line) and a simulation where otolith opacity depends only on temperature (magenta, dashed-dotted line). The correlation coefficients with the real data were R2 = 0.90, R2 = 0.66 and R2 = 0.43, respectively (p<0.001 in all cases). (TIF) [file pone.0027055.s004.tif]

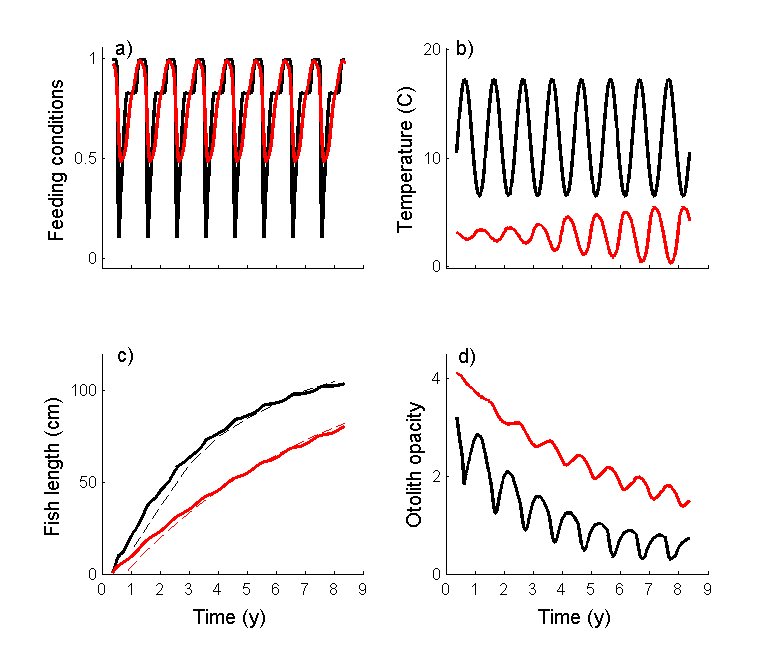

Supplement: Figure S5 — Model simulations for Southern North Sea cod (NS, black) and Barents Sea cod (BS, red): food density series (a), temperature series (b), somatic growth patterns (c), and otolith opacity patterns (d). The somatic growth data (panel c, dashed lines) were obtained from Bolle et al. (Jørgensen 1992) for the both populations. (TIF) [file pone.0027055.s005.tif]

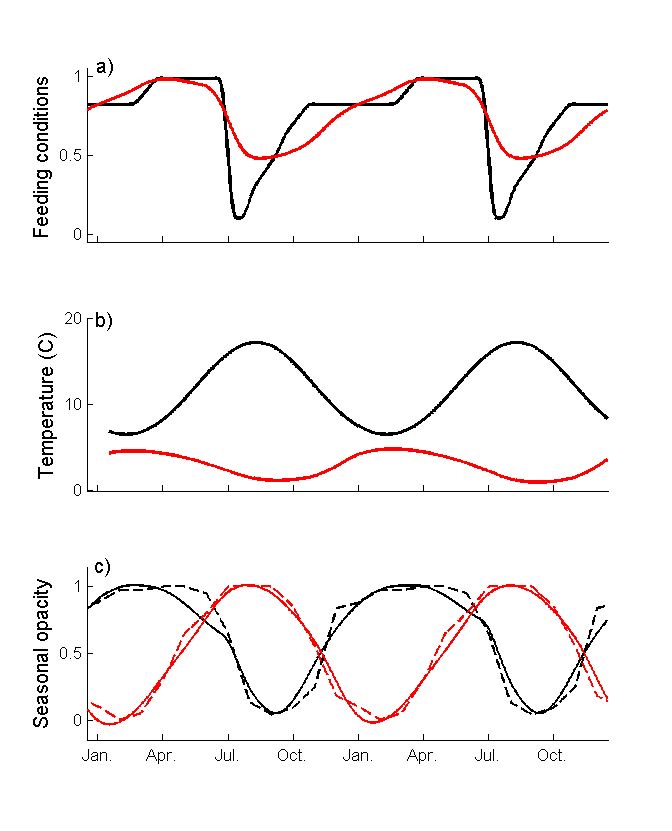

Supplement: Figure S6 — Seasonality of the timing of otolith zone formation for the simulated and real data for NS and BS cod: feeding conditions (a), temperature conditions (b), and seasonal opacity patterns (c). BS cod are represented by red and NS cod by black. We compared the average proportions of translucent otolith edges for real otoliths taken from Høie et al. (Høie, Millner et al. 2009) (dashed lines) to identify simulated seasonal opacity patterns (solid lines). (TIF) [file pone.0027055.s006.tif]

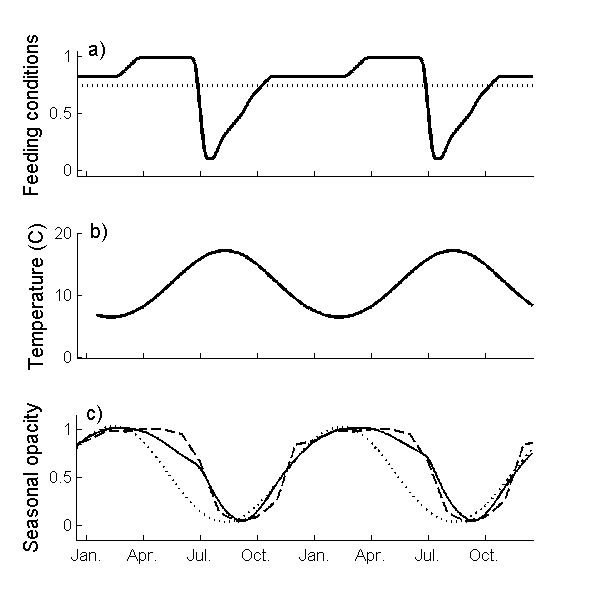

Supplement: Figure S7 — Seasonal otolith opacity patterns for NS cod with constant and non-constant feeding conditions: feeding conditions (a), temperature conditions (b), and seasonal opacity patterns (c). We display two simulations: the one reported in Fig. S6 (solid lines, R2 = 0.96, p>0.001) and a scenario assuming a constant feeding with the temperature conditions used in Fig. S6 (dotted lines, R2 = 0.64, p>0.001). Simulated opacity patterns are compared to the otolith data (dashed, see Fig. S5). (TIF) [file pone.0027055.s007.tif]

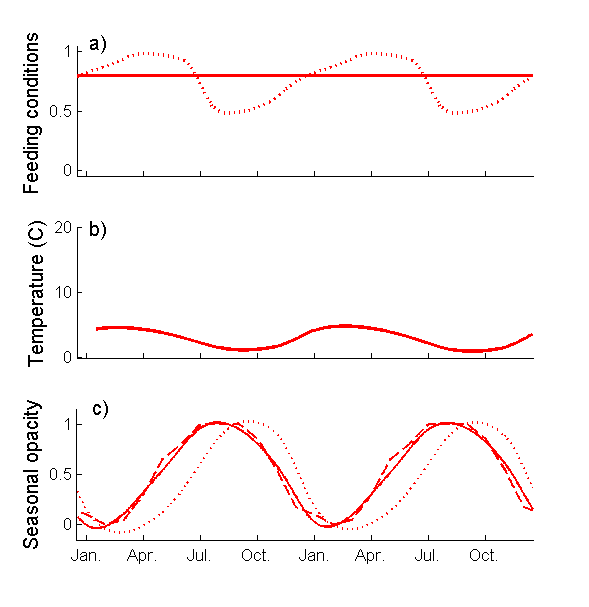

Supplement: Figure S8 — Seasonal otolith opacity patterns for BS cod with constant and non-constant feeding conditions: feeding conditions (a), temperature conditions (b), and seasonal opacity patterns (c). We display two simulations: the one reported in Fig. S6 (solid lines, R2 = 0.96, p>0.001) and a scenario assuming a constant feeding with the temperature conditions used in Fig. S6 (dotted lines, R2 = 0.54, p>0.001). The simulated opacity patterns are compared to the real otolith data (dashed line, see Fig. S5). (TIF) [file pone.0027055.s008.tif]

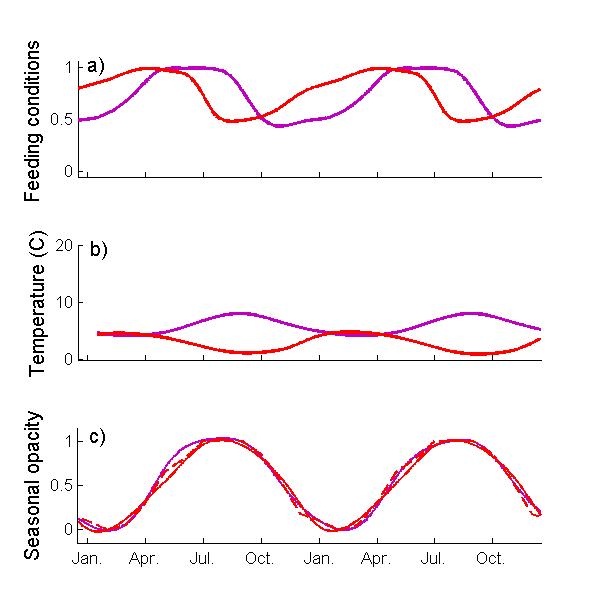

Supplement: Figure S9 — Seasonality of the timing of otolith zone formation for BS and Norwegian coastal (NC) cod: feeding conditions (a), temperature conditions (b) and seasonal opacity patterns (c). BS cod are shown in red and NC cod in magenta. Both populations are known to display the same seasonal otolith opacity pattern. We compared the average proportions of translucent otolith edges for real otoliths taken from Høie et al. (Høie, Millner et al. 2009) (dashed lines) with simulated seasonal opacity patterns (solid lines). (TIF) [file pone.0027055.s009.tif]

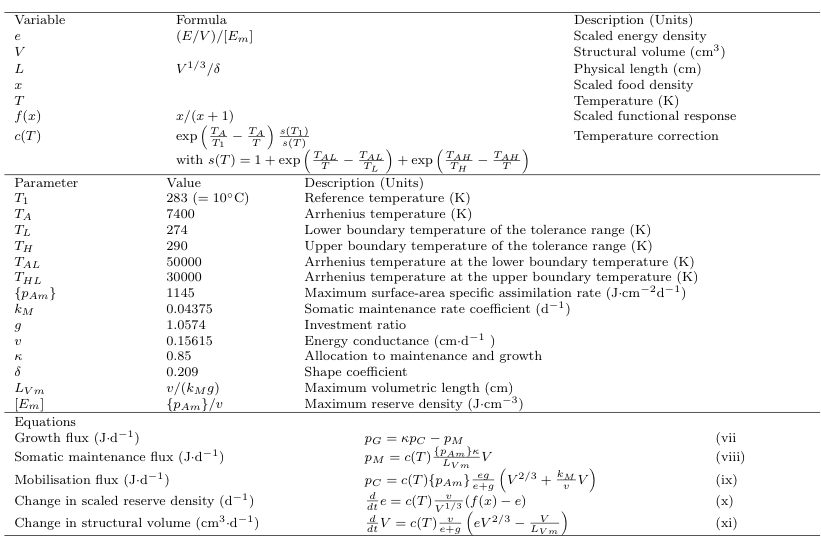

Supplement: Table S1 — Variables, parameter values and equations for individual growth and somatic maintenance in a standard DEB model. (TIFF) [file pone.0027055.s011.tif]

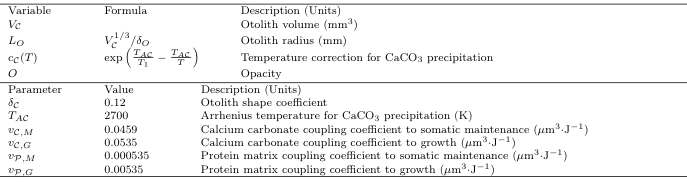

Supplement: Table S2 — Variables, parameter values and equations for otolith biomineralization. (TIFF) [file pone.0027055.s012.tif]
