# Supplementary material for: Shedding Light on Fish Otolith Biomineralization Using a Bioenergetic Approach
Source: PLoS One. 2011 Nov 14;6(11):e27055. doi: 10.1371/journal.pone.0027055 (PMC3215717; doi:10.1371/journal.pone.0027055)
Supplement: Text S1 — This Supplementary Text provides further details and analysis regarding the key aspects of the proposed bioenergetic model of otolith biomineralization.It is organized as a report and involves three main sections: 1. A generic bioenergetic model of otolith biomineralization. This section further details model assumptions and equations. 2. Model calibration and validation. This section details calibration and validation dataset and results, and report calibrated model parameters. 3. Resolving the seasonal timing of the formation of opaque and translucent zones in fish otoliths of different cod populations. This section details the analysis, from model simulations, of the non-synchronous and synchronous seasonal opacity otolith patterns of several cod populations, namely Barrents Sea, Southern North Sea and Norwegian coast cod populations. (DOC) [file pone.0027055.s010.doc]

**Supporting Information for ‘Shedding light on fish otolith biomineralization using a bioenergetic approach’** by FABLET, R., PECQUERIE, L., de PONTUAL, H., HOIE, H., MILLNER R., MOSEGAARD, H., and KOOIJMAN, S.AL.M.

**Summary of the Supplementary Text S1**

This Supplementary Text provides further details and analysis regarding the key aspects of the proposed bioenergetic model of otolith biomineralization. It is organized as a report and involves three main sections:

- **1. A generic bioenergetic model of otolith biomineralization**. This Supp. Mat. further details model assumptions and equations.
- **2. Model calibration and validation.** This Supp. Mat. details calibration and validation dataset and results, and report calibrated model parameters.
- **3. Resolving the seasonal timing of the formation of opaque and translucent zones in fish otoliths of different cod populations.** This Supp. Mat. details the analysis, from model simulations, of the non-synchronous and synchronous seasonal opacity otolith patterns of several cod populations, namely Barrents Sea, Southern North Sea and Norwegian coast cod populations.

**List of Tables**

**Table S1. Variables, parameter values and equations for individual growth and somatic maintenance in a standard DEB model.**

**Table S2. Variables, parameter values and equations for otolith biomineralization.**

**List of Figures of the Supplementary Material**

**Figure S1. Model simulations for a shift in feeding conditions (Exp. 1)**. This figure reports temperature and feeding conditions in Experiment 1 and comparison between model simulations and real otolith data for fish growth and otolith growth.

**Figure S2. Simulations of opacity patterns for a shift in feeding conditions (Exp. 1)**. This figure compares model simulations to real opacity data for Exp.2 for different model assumptions.

**Figure S3. Model simulation for constant feeding conditions and seasonal temperature cycles (Exp. 2).** This figure reports temperature and feeding conditions in Experiment 2 and comparison between model simulations and real otolith data for fish growth and otolith growth.

**Figure S4. Simulation of opacity patterns for constant feeding conditions and seasonal temperature cycles (Exp. 2).** This figure compares model simulations to real opacity data for Exp.2 for different model assumptions.

**Figure S5: Model simulations for Southern North Sea cod and Barents Sea cod.** This figure depicts the temperature and feeding scenarios considered for the two cod populations as well as the associated simulations of fish growth and otolith opacity.

**Figure S6: Seasonality of the timing of otolith zone formation for the simulated and real data for Southern North Sea and Barents Sea cod.** This figure reports the real and simulated seasonal opacity patterns for the two cod populations.

**Figure S7: Seasonal otolith opacity patterns for real Southern North Sea cod with constant and non-constant feeding conditions.** This figure compares the simulated opacity patterns for two different feeding condition scenarios to the observed opacity pattern for the Southern North Sea cod population.

**Figure S8: Seasonal otolith opacity patterns for Barents Sea cod with constant and non-constant feeding conditions.** This figure compares the simulated opacity patterns for two different feeding condition scenarios to the observed opacity pattern for the Barents Sea cod population.

**Figure S9: Seasonality of the timing of otolith zone formation for Barents Sea and Norwegian coast cod populations.** This figure reports the real and simulated seasonal opacity patterns for the two cod populations.

We first present the conceptual model for otolith biomineralization that we developed within the framework of the Dynamic Energy Budget (DEB) theory. We then describe the calibration and the validation of this model using experimental otolith data. And, we detail how we applied this model to resolve the non-synchronous seasonal timing of the formation of opaque zones in the otoliths of two cod populations in the North Sea and the Barents Sea.

# A generic bioenergetic model of otolith biomineralization

## Motivation and model assumptions

Fish otoliths are located in the inner ears of fish and are involved in balance regulation (Popper 1976). They are mainly composed of aragonite, a calcium carbonate polymorph. Their shape is species-specific, but otolith formation depicts generic properties that are widely used in the biological sciences for reconstruction of fish age, growth, natal origin and migration patterns (Campana 2001; Elsdon, Wells et al. 2008). Similar to the rings of a tree trunk, the otoliths present opaque and translucent bands from which the age of an individual can be determined (Campana 2001). Moreover, as the size of the otolith is often highly correlated to fish length, otolith growth between two bands has been widely used to reconstruct fish growth during that period (Campana 1990).

Numerous studies have shown that bias and uncertainty are associated with otolith interpretations, however (Campana 1990; Beamish and McFarlane 1995; Beckman and Wilson 1996). Opaque and translucent bands may form at irregular time intervals or may not be distinguishable(de Pontual, Groison et al. 2006; Høie, Millner et al. 2009), which has led to erroneous conclusions due to misinterpretation of the age implications of the different bands (Beamish and McFarlane 1995; Reeves 2003; Andrews, Tracey et al. 2009) Otolith size is also not simply proportional to fish length, which may result in biased growth reconstruction (Campana 1990). Although there are methods to correct for these uncertainties, a general theoretical and mechanistic framework for the mechanisms controlling both otolith opacity and growth is lacking. Recent experimental studies have addressed this issue but they were not able to decipher the complex interplay between metabolic and environmental factors and seemingly contradictory conclusions were reported(Høie, Folkvord et al. 2008; Neat, Wright et al. 2008).

From a physical-chemical point of view, biomineralization is described by the precipitation equation

Ca^2+^ + HCO_3_^-^ ⇔ Ca CO_3_ + H^+^ (i),

which is characterized by its saturation coefficient Ω = [Ca^2+^]. [HCO_3_^-^]K_s_^-1^, where [Ca^2+^] and [HCO_3_] are the activity of calcium and carbonate ions, respectively, in the endolymph at the otolith surface (body text, Fig. 1), and K_S_ is the solubility product[[1]](#footnote-2). The value of the saturation coefficient Ω characterizes the saturation state of the solution and should be larger than 1 if precipitation is to occur.

A purely mineral setting, as exploited for instance by Romanek and Gauldie (Romanek and Gauldie 1996), fails to capture other well-known otolith properties that have been linked to fish metabolism, however. The growth rate of an individual impacts the size of its otoliths at a given length; slow-growing fish have larger otoliths when compared to fast-growing fish of the same length, and temperature generally increases otolith growth rate (Mosegaard, Svedäng et al. 1988). Otolith growth has also been correlated to respiration rate (Wright 1991; Yamamoto, Ueda et al. 1998). A decreasing trend in opacity as a fish is growing has also been generally observed (Hussy, Mosegaard et al. 2004). Recent studies have provided a better understanding of otolith formation at the precipitation site by showing how the organic matrix in the otolith and the different organic compounds present in the endolymph surrounding the otolith control its shape and growth (Allemand, Mayer-Gostan et al. 2007). Otolith opacity has also been shown to correlate to the major organic fractions (Mugiya 1965; Hussy, Mosegaard et al. 2004). However, there is a gap in the understanding of how these detailed metabolism-driven processes relate to the varying nutritional conditions of the fish and ambient environmental conditions.

We here propose a simple model linking variations in opacity and growth of an otolith to variations in metabolism as a fish grows and experiences varying environmental conditions. The Dynamic Energy Budget (DEB) theory (Kooijman 2010) provides a quantitative framework for this study that is based on first principles. This general theory for metabolic organization describes how an organism assimilates and utilizes energy for maintenance, growth, development and reproduction throughout its life cycle. It has been successfully applied to many taxa, including fish (Bodiguel, Maury et al. 2009; Pecquerie, Petitgas et al. 2009). The key step in this study is the application of the DEB concept to the otolith stated as a metabolic product.

Because otoliths do not require maintenance and are not mobilized to fuel other metabolic processes, otoliths do not relate directly to two generalized compounds, structure and reserve, that the DEB theory delineates to specify assimilation, growth, development and maintenance processes in an individual. Compounds that do not observe these two definitions are defined as metabolic products. These compounds can be excreted into the environment (e.g., feces, CO_2_, and NH_3_) but can also be part of the total biomass (e.g., tree trunks and mammalian hairs). The rate at which they are formed is controlled by the metabolism of the organism. This rate is stated as a weighted sum of the three main processes: assimilation processes, growth processes and dissipation processes, such as somatic maintenance or development. Some of these coefficients may be zero (e.g. feces has contributions from assimilation but not from growth or somatic maintenance).

Formally, the application of the concept of the metabolic DEB product to fish otoliths exploits a standard DEB model for fish growth, and we supplemented our model of otolith formation with the following assumptions (body text, Fig. 1):

*A1. Both the aragonite fraction and the matrix proteins of an otolith are metabolic products.*

We consider an otolith composed of two fractions: a mineral fraction (95-99.9% of the otolith volume) and an organic fraction (0.1-5% of the volume). This representation is more detailed than others (Pecquerie 2008) to introduce a specific temperature effect on CaCO_3_ precipitation and more detailed mechanisms for opacity variations (see the following assumptions).

*A2. Growth and somatic maintenance fluxes contribute to the formation of both the mineral and the organic fractions of an otolith. Assimilation and other dissipated fluxes have no contribution.*

The latter part of the assumption is motivated by the observation that otoliths are already growing in embryos, which are not assimilating food.

*A3. The dynamics of CaCO_3_ precipitation is temperature-dependent.*

In vitro experiments have shown that temperature directly affects aragonite precipitation dynamics (Romanek and Gauldie 1996). These dynamics are described by a power law, λ(Ω-1)^γ^, with parameters λ and γ. The greater growth rate of the crystal as temperature increases is captured by the temperature-dependent parameter λ. We further assume that the temperature within the saccular epithelium equals that of the water.

*A4. Variations in opacity of the newly precipitated volume reflect variations of the organic fraction relative to the mineral fraction at a macroscopic level.*

We focus on the macrostructure level and do not consider the processes operating on a daily scale that induce variations in opacity at a microstructure level (Panella 1971). At the macrostructure level, otolith opacity has been positively correlated to the amount of protein deposited (Hussy, Mosegaard et al. 2004).

*A5. Otolith growth is isomorphic and the organic fraction contributes little to the total volume.*

Because 95% to 99% of the otolith volume is aragonite, we assume that the organic fraction can be neglected.

## Equations and model properties

Assumptions A1, A2 and A3 imply that both the volume of the organic matrix *V_P_* (µm^3^) and the volume of aragonite *V_C_* (µm^3^) increase as a weighted sum of the growth flux p_G_ and the somatic maintenance flux p_M_ (SI, Table S1):

(ii)

$\frac{dV_{P}}{dt}=\alpha_{P}p_{G}+\beta_{P}p_{M}$ (iii),

where α_C_, β_C_, α_P_, β_P_ (µm^3^.J^-1^) are the coupling coefficients and factor *c_C_(T)* results from the direct effect of temperature on CaCO_3_ precipitation (A3). More precisely, assuming a power law λ(Ω-1)^γ^ for the precipitation dynamics of the mineral fraction with a temperature-dependent parameter λ, a first-order approximation leads to the parameterization proposed in Eq.(ii) for the dynamics of the mineral fraction of the otolith as a DEB metabolic product, where temperature-driven factor *c_C_(T)* quantifies the change, w.r.t. a reference temperature, in the precipitated mineral volume for given metabolic conditions. Given temperature-related in vitro aragonite precipitation dynamics (Romanek and Gauldie 1996), factor *c_C_(T)* is an increasing function of T. Here, a parameterization with an Arhenius law was considered.

The opacity value, *O,* of the newly precipitated volume is given by the proportion of the volume ΔV_P_ of matrix proteins relative to the volume ΔV_C_ of calcium carbonate precipitated (A4):

$O=\frac{\Delta V_{P}}{\Delta V_{C}}\approx\frac{\frac{dV_{P}}{dt}}{\frac{dV_{C}}{dt}}=\frac{1}{c_{C}(T)}\cdot\frac{\alpha_{P}p_{G}+\beta_{P}p_{M}}{\alpha_{C}p_{G}+\beta_{C}p_{M}}$ (iv)

We assume here that the volume of the matrix proteins contributes little to the otolith radius *L_O_* (mm) (A5), i.e., that

$L_{O}=\frac{V_{C}^{1/3}}{\delta_{O}}$ (v),

where δ_O_ is a shape coefficient.

The proposed model reproduces several well-known properties of otolith growth:

1. a high correlation between fish length and otolith size (Campana 1990) that is seen when the contribution from growth dominates (i.e., α_C_ *p_G_ >>* β_C_*p_M_*);
2. a decoupling between fish length and otolith size (Campana 1990) that occurs as soon as the contribution from somatic maintenance becomes substantial (e.g., during non growing periods, somatic maintenance still occurs). Because the contribution from growth is the same for fish of the same length but the contribution from maintenance occurs over a longer period of time for slower growing individuals (resulting in larger otolith sizes); this mechanism explains why slow-growing fish have larger otoliths than fast-growing ones of the same length;
3. a correlation between otolith growth and specific rates of oxygen consumption because the DEB theory implies that oxygen consumption can also be stated as a weighted sum of assimilation, growth and dissipation processes (i.e., a function of DEB metabolic fluxes similar to otolith growth) (Wright 1991; Yamamoto, Ueda et al. 1998).

The model also reproduces the following known otolith opacity patterns.

1. a decrease in opacity as the fish approaches its asymptotic length (Hoff and Fuiman 1995; Hussy and Mosegaard 2004);
2. a reduction of the feeding condition resulting in a decrease of the somatic growth flux p_G_, which then leads to the accretion of more translucent material (Høie, Folkvord et al. 2008) if the contribution of the somatic growth flux is greater in the organic matrix flux than in the calcium carbonate flux (i.e., $\frac{\alpha_{P}}{\beta_{P}}>\frac{\alpha_{C}}{\beta_{C}}$). Conversely, more opaque otolith material is deposited when food availability increases;
3. temperature variations that directly affect CaCO_3_ precipitation rates more than organic flux, resulting in higher opacity as a result of colder waters (Høie and Folkvord 2006; Neat, Wright et al. 2008).

Furthermore, simultaneous variations in feeding and temperature conditions can have joint or opposite effects on otolith growth and opacity. It should, however, be noted that these two effects differ in their time response. Whereas from relationships (ii) and (iv) a shift in temperature has an immediate effect on otolith growth and opacity, a variation in feeding conditions may not be perceived immediately. A decrease in feeding conditions is buffered by the reserve, and the associated time constant could typically range from a few days to a few months, depending on the size of the individual, its condition and the temperature it experiences (Kooijman 2010).

As illustrated in the following section, these properties provide the basis for explaining the complexity of the observed otolith patterns with respect to fish metabolism and experienced environmental conditions.

Table S1: Variables, parameter values and equations for individual growth and somatic

maintenance in a standard DEB model.

Table S2: Variables, parameter values and equations for otolith biomineralization.

## 2D simulations

To simulate otolith images and compare the simulated images with real ones, we extended our otolith-DEB model to 2D using the shape model of Fablet et al. (Fablet, Pujolle et al. 2008; Fablet, Chessel et al. 2009). We first build a reference otolith shape model from a collection of images of otoliths of the species and/or stock in question. We form an otolith image by evolving this shape model according to the growth rate specified by the otolith-DEB model and assigning the associated opacity value to the corresponding 2D growth increment. We also account for the spatial anisotropy of otolith opacity (Fablet, Chessel et al. 2009) as follows:

$O_{s}\left(p(t)\right)=O(t)+\alpha\cdot GA\left(p(t)\right)$ (vi),

where p(t) is an image pixel in the 2D growth increment formed at time t, O(t) is the opacity value predicted by the otolith-DEB model, GA(p(t)) is the growth anisotropy factor at pixel p(t) (Fablet, Chessel et al. 2009) and α is a parameter, typically set to 5.

# Model calibration and validation

## Experimental data

To calibrate and validate the model, we used data from two different experiments. In Experiment 1, the impact of a shift in feeding conditions was examined (Høie, Folkvord et al. 2008). One-year-old fish ranging from 30 to 35 cm were reared in high feeding conditions for 100 days, lower feeding conditions for the subsequent 120 days and *ad libitum* conditions for the last 80 days. The fish experienced seasonal temperature variations, with a seasonal high at day 60 and a seasonal low at day 250. In Experiment 2, the cods were 7 months old at the start of the experiment. They were fed *ad libitum* for 22 months and experienced seasonal temperature conditions (IBACS 2006). In both cases, calibrated otolith data (i.e., time-referenced otolith growth[[2]](#footnote-3) and opacity data) are available along with the fish growth data.

## Model calibration and validation

We calibrated the fish growth model using the parameter values specified in Table S1 (van der Veer, Freitas et al. 2010) and the length data from Exp. 1. The parameters of the Arrhenius law for the temperature modulation of DEB fluxes were obtained from the temperature-related cod growth data reported by Björnsson et al. (Björnsson, Steinarsson et al. 2007). We also calibrated the otolith growth and opacity model using otolith data from Exp. 1 (SI, Tab. S2). Predicted opacity patterns were compared to real ones using correlation statistics, and the associated regression was used to visually check the consistency between the real and simulated opacity signals. We validated the model by comparing the model simulations to the otolith data of Exp. 2.

The model simulations of fish and otolith growth in response to a shift in feeding regimes (SI, Fig. S1) depicted, as expected, a decrease in growth rates during the low-feeding period, but with a lower effect on otolith growth. A good match between the simulations and the real otolith and fish growth was found. The simulation without the temperature-specific effect (i.e., with parameter T_AC_ set to 0 so that regulation c_C_(T) equals 1 in relationships (ii) and (iv)) recovered the general opacity trend with an abrupt opacity decrease after the shift to the low-feeding period. A better correlation between the real opacity pattern and the predicted one (R^2^ = 0.96 vs. 0.93) was obtained when the temperature-specific effect was introduced (SI, Fig. S2). This example is a good illustration of the joint effect of feeding and temperature conditions on otolith opacity; while the opacity trend is due to the shift in the feeding variations, the higher-frequency variations of the opacity are explained by temperature variations. For example, it can be noted that the temperature peak at day 60 corresponds to a relative minimum of the opacity (SI, Fig. S2).

[SI, Figures S1 & S2]

Given the model calibrated from Exp. 1, we performed model simulations for Exp. 2. Temperature and feeding conditions, as well as the comparison of the predicted and observed fish and otolith length, are reported (SI Fig. S3).

Regarding the opacity pattern, we further illustrated the relevance of the proposed model with a comparison of the simulation of the calibrated model for Exp. 2 to two other simulations (SI, Fig. S4):

- 1) a simulation ignoring the otolith-specific temperature effect (i.e., the parameter T_AC_ was set to 0 so that c_C_(T) equaled 1 over the entire temperature range);
- 2) a simulation with otolith opacity as a direct function of temperature. We considered a linear function of the inverse regulation factor c_C_(T) (i.e., in relationship (iii), otolith parameters v_P,G_ and v_P,M_ are set such that $v_{P,G}/v_{P,M}=v_{C,G}/v_{C,M}$) to cancel the metabolism-driven effect.

[SI, Figure S3]

These following observations on the simulations are worth noting:

1. the opacity variations can be mostly explained by temperature variations introduced into the model by the otolith-specific temperature-regulated factor c_C_(T), which resulted in a correlation coefficient greater than 0.9 (p <1e-5) compared to a correlation coefficient below 0.7 when this temperature effect was not incorporated;
2. it should, however, be pointed out that this 800-day experiment depicts a decreasing opacity trend that stresses the relevance of the opacity model that incorporates joint temperature- and metabolism-driven effects. Consequently, temperature alone, through factor c_C_(T), cannot explain the observed patterns (R^2^=0.9 vs. R^2^<0.5).

[SI, Figure S4]

# Resolving the seasonal timing of the formation of opaque and translucent zones in fish otoliths of different cod populations

## Objective

Meta-analyses have highlighted contradictory conclusions among species and populations in the analysis of the seasonality of otolith opacity patterns in relation to environmental conditions (Beckman and Wilson 1996; Campana 2001). Translucent[[3]](#footnote-4) zones are generally considered to be formed during slow-growing winter periods and opaque zones during fast-growing spring and summer periods. However, this hypothesis has been proven to be incorrect in numerous cases (Williams, Davies et al. 2005; Høie, Millner et al. 2009), and there is no unifying conceptual framework for solving these contradictions.

Southern North Sea cod (NS) and Barents Sea cod (BS) populations are peculiar examples. Their otolith patterns depict non-synchronous seasonal opacities (Høie, Millner et al. 2009) Whereas BS cod otoliths follow the general pattern with the formation of a translucent zone in winter (December-April), in NS cod otoliths the translucent zone is deposited in summer-early fall (July-November), which is five months earlier than in the Barents Sea fish. We apply our otolith-DEB model to these two populations and show that we can solve the seemingly contradictory seasonal opacity patterns of these two populations from the differences in the environments experienced by the fish.

Given the calibrated otolith-DEB detailed in the previous section, an analysis of the seasonal otolith opacity patterns of different cod populations is carried out as described below.

## Resolving the non-synchronous seasonality of the otolith opacity patterns of BS and NS cod populations

We investigated two different environmental scenarios to understand the seemingly contradictory seasonal otolith opacity patterns in North Sea (NS) and Barents Sea (BS) cod:

- 1) for the NS cod population, the yearly temperature conditions follow the dynamics of surface temperatures in the southern North Sea. Following (Pilling, Millner et al. 2007), a low feeding behaviour corresponding to temperature highs from August to October is considered in contrast to mid-level feeding conditions from December to February and high-level feeding conditions between March and July.
- 2) BS cods undertake a long seasonal migration (Godø and Michalsen 2000). During the summer, cod feed in the northern part of the Barents Sea, at the ice border, but migrate southward in wintertime. This migratory behavior results in an inverse temperature cycle: the fish experience cooler summer temperatures and warmer winter temperatures. The range of the seasonal migration increases with the age of the fish so that older fish experience greater temperature gradients. The temperature conditions considered in the model simulations were based on records of data storage tags (Godø and Michalsen 2000). In accord with the seasonal migration, a peak in feeding conditions was assumed from March to April, corresponding to the seasonal feeding on capelin (Yaragina and Marshall 2000), followed by seasonal August-to-November low feeding conditions prior to the start of the southward migration in December.

We used model parameters calibrated from Exp. 1 for the two populations. The mean feeding condition was set to 0.75 for the BS stock and 0.8 for the NS cod stock to fit the simulated somatic growth patterns to the data reported in the literature (Bolle, Rijnsdorp et al. 2004) (SI, Fig. S5). It should also be noted that the ranges of both temperature and feeding conditions are smaller for BS cod than for NS cod.

[SI, Figure S5]

The model simulations were compared to the otolith data reported in by (Høie, Millner et al. 2009), i.e. the monthly proportions of opaque otolith edges. Normalized seasonal opacity predictions were computed as detrended versions of the predicted opacity signals, rescaled between 0 and 1. The seasonal patterns were extracted for age classes 2 and 3 for the NS cod and age classes 4 and 5 for comparison with the sampled fish data (Høie, Millner et al. 2009). Model simulations based on the proposed forcing scenarios reproduced the seasonal patterns observed for both populations (SI, Fig. S6, R^2^ = 0.97). The simulated images are similar to the real ones, especially with regard to the greater contrast in NS cod otolith images resulting from the greater range of experienced temperature and feeding conditions (body text, Fig. 3C). For a dynamic visualization of the differences in the timing of the formation of seasonal otolith structures, animated versions of the model predictions are included as electronic appendices (SI, Video S1). Interestingly, the seasonal opacity patterns are better explained by the combined temperature and feeding scenarios (SI, Fig. S6, R^2^ > 0.96), rather than scenarios where temperature conditions alone vary (SI, Fig. S7 & S8, R^2^ < 0.65). It should be noted, however, that we resorted to different scenarios of the formation of the seasonal translucent zone for the two populations; whereas the translucent zone for the NS cod stock is formed in late summer due to conjunction of temperature highs and a low feeding behaviour, the translucent zone deposited in the winter for the BS cod mainly appears as a result of the warmer temperatures experienced by the fish, with a seasonal peak in March. Beyond seasonal opacity patterns, the model also predicts greater opacity values and rather poorly contrasted otoliths for the BS cod stock as a consequence of the significantly lower seasonal temperature and feeding gradient experienced by the fish.

[SI, Figures S6, S7 & S8]

As observed for the BS and NS cod populations, differences in the seasonal environmental conditions experienced by fish may result in non-synchronous seasonal otolith opacity patterns. However, fish populations with the same seasonal otolith opacity patterns may not share similar environmental conditions. For example, the Norwegian coastal (NC) cod stock displays seasonal otolith opacities similar to those of the BS cod stock (Høie, pers. comm.) but does not perform a long winter migration and experiences colder temperatures in winter and warmer temperatures in summer. Our model simulations[[4]](#footnote-5) (SI, Fig. S9, R^2^ > 0.98) show that feeding highs from April to August and lows from October to February result in a seasonal opacity pattern for the NC cod stock synchronous with that of the BS cod stock.

[SI, Figure S9]

These examples outline the apparent complexity of the formation of otolith zones and illustrate how first principle mechanisms for the biomineralization of the otolith provide an understanding of differences and/or similarities in otolith patterns among populations or species from knowledge of fish behavior and environment.

References

Allemand, D., N. Mayer-Gostan, et al. (2007). Fish Otolith Calcification in Relation to Endolymph Chemistry. Handbook of biomineralization. E. Bäuerlein, Behrens, P., and Epple, M. (eds.), Wiley. **1:** 291-308.

Andrews, A. H., D. M. Tracey, et al. (2009). "Lead–radium dating of orange roughy (Hoplostethus atlanticus): validation of a centenarian life span." Canadian Special Publications in Fisheries and Aquatic Sciences **66**(7): 1130-1140.

Beamish, R. J. and G. A. McFarlane (1995). A discussion of the importance of aging errors, and an application to walleye pollock: the world's largest fishery. Recent Developments in Fish Otolith Research (Secor, D. H., Dean, J. M. Campana, S. E., ). Columbia University of South Carolina Press**:** 545–565.

Beckman, D. W. and A. W. Wilson (1996). Seasonal timing of opaque zone formation in fish otoliths. Recent developments in fish otolith research. D. H. Secor, Dean, J.M., Campana, S.E., Univ. of South Carolina Press**:** 27-43.

Björnsson, B., A. Steinarsson, et al. (2007). "Growth model for Atlantic cod (Gadus morhua): Effects of temperature and body weight on growth rate." Aquaculture **271**(1-4): 216-226.

Bodiguel, X., O. Maury, et al. (2009). "A dynamic and mechanistic model of PCB bioaccumulation in the European hake (Merluccius merluccius)." Journal of Sea Research **62**(2-3): 124-134.

Bolle, L. J., A. D. Rijnsdorp, et al. (2004). "Growth changes in plaice, cod, haddock and saithe in the North Sea: a comparison of (post-)medieval and present-day growth rates based on otolith measurements." Journal of Sea Research **51**(3-4): 313-328.

Campana, S. E. (1990). "How Reliable are Growth Back-Calculations Based on Otoliths?" Canadian Journal of Fisheries and Aquatic Sciences **47**(11): 2219–2227.

Campana, S. E. (2001). "Accuracy, precision and quality control in age determination, including a review of the use and abuse of age validation methods." Journal of Fish Biology **59**(2): 197-242.

de Pontual, H., A. L. Groison, et al. (2006). "Evidence of underestimation of European hake growth in the Bay of Biscay, and its relationship with bias in the agreed method of age estimation." ICES Journal of Marine Science **63**(9): 1674-1681.

Elsdon, T. S., B. K. Wells, et al. (2008). "Otolith chemistry to describe movements and life-history measurements of fishes: hypotheses, assumptions, limitations, and inferences using five methods." Oceanography and Marine Biology: an Annual Review **46**: 297-330.

Fablet, R., A. Chessel, et al. (2009). "Reconstructing individual shape histories of fish otoliths: a new image-based tool for otolith growth analysis and modeling." Fisheries Research **96**(2-3): 148-159.

Fablet, R., S. Pujolle, et al. (2008). "2D Image-based reconstruction of shape deformation of biological structures using a level-set representation." Computer Vision and Image Understanding **111**(3): 295-306.

Godø, O. R. and K. Michalsen (2000). "Migratory behaviour of north-east Arctic cod, studied by use of data storage tags." Fisheries research **48**(22): 127-140.

Hoff, G. R. and L. A. Fuiman (1995). " Environmentally-induced variation in elemental composition of red drum (Sciaenops-ocellatus) otoliths." Bulletin of Marine Science **56**(2): 578-591.

Høie, H. and A. Folkvord (2006). "Estimating the timing of growth rings in Atlantic cod otoliths using stable oxygen isotopes." Journal of Fish Biology **68**(3): 826-837.

Høie, H., A. Folkvord, et al. (2008). "Restricted fish feeding reduces cod otolith opacity." Jal of Applied Ichtyology **24**: 138-143.

Høie, H., R. Millner, et al. (2009). "Latitudinal differences in the timing of otolith growth: A comparison between the Barents Sea and southern North Sea." Fisheries Research **96**(2-3): 319-322.

Hussy, K. and H. Mosegaard (2004). "Atlantic cod (Gadus morhua) growth and otolith accretion characteristics modelled in a bioenergetics context." Canadian Journal of Fisheries and Aquatic Sciences **61**(6): 1021-1031.

Hussy, K., H. Mosegaard, et al. (2004). "Effect of age and temperature on amino acid composition and the content of different protein types of juvenile Atlantic cod (Gadus morhua) otoliths." Canadian Journal of Fisheries and Aquatic Sciences **61**(6): 1012-1020.

IBACS (2006). WP6 report: Long time rearing for monitoring of near natural conditions. IBACS project (Integrated Approach to the Biological Basis of Age Estimation in Commercially Important Fish Species, QQLRT-2001-01610).

Jørgensen, T. (1992). "Long-term changes in growth of North-east Arctic cod (Gadus morhua) and some environmental influences." ICES J. Mar. Sci. **49**(3): 263-278.

Kooijman, S. A. L. M. (2010). Dynamic Energy Budget theory for Metabolic Organisation, Cambridge University Press.

Mugiya, Y. (1965). "Calcification in fish and shell-fish-IV. The difference in nitrogen content between the translucent and opaque zone of otolith." Bull. Jpn. Soc. Sci. Fish. **31**: 896-901.

Neat, F. C., P. Wright, et al. (2008). "Temperature effects on otolith pattern formation in Atlantic cod Gadus morhua." Jal Fish Biology **73**(10): 2527 - 2541.

Panella, G. (1971). "Fish otoliths: daily growth layers and periodical patterns." Science **173**(2): 1124-7.

Pecquerie, L. (2008). Bioenergetic modelling of the growth, development and reproduction of a small pelagic fish: the Bay of Biscay anchovy, Vrije Universiteit. **PhD**.

Pecquerie, L., P. Petitgas, et al. (2009). "Modeling fish growth and reproduction in the context of the Dynamic Energy Budget theory to predict environmental impact on anchovy spawning duration." Journal of Sea Research **62**(2-3): 93-105.

Pilling, G. M., R. S. Millner, et al. (2007). "Phenology and North Sea cod Gadus morhua L.: has climate change affected otolith annulus formation and growth?" Journal of Fish Biology **70**(2): 584–599.

Popper, A. N. (1976). "Ultrastructure of the auditory regions in the inner ear of the lake whitefish." Science **192**(4243): 1020-1023.

Reeves, S. A. (2003). "A simulation study of the implications of age-reading errors for stock assessment and management advice." ICES J. Mar. Sci. **60**(2): 314-328.

Romanek, C. S. and R. W. Gauldie (1996). "A predictive model of otolith growth in fish based on the chemistry of the endolymph." Comparative Biochemistry and Physiology a-Physiology **114**(1): 71-79.

van der Veer, H., V. Freitas, et al. (2010). "Temperature tolerance and energetics, a Dynamic Energy Budget-based comparison of North Atlantic marine species. ." Philosophical Transactions of the Royal Society B: Biological Sciences. Special issue "Developments in Dynamic Energy Budget theory and its applications".

Williams, A. J., C. R. Davies, et al. (2005). "Variation in the periodicity and timing of increment formation in red throat emperor (Lethrinus miniatus) otoliths " Marine and Freshwater Research **56**(5): 529-538.

Wright, P. J. (1991). "The influence of metabolic rate on otolith increment width in Atlantic salmon parr, Salmo salar L." Journal of Fish Biology **38**: 929-933.

Yamamoto, T., H. Ueda, et al. (1998). "Correlation among dominance status, metabolic rate and otolith size in masu salmon." Journal of Fish Biology **52**(2): 281-290.

Yaragina, N. A. and C. T. Marshall (2000). "Trophic influences on interannual and seasonal variation in the liver condition index of Northeast Arctic cod (Gadus morhua)." ICES J. Mar. Sci. **57**: 42-55.

1. The solubility product is defined as the product of the activities of calcium and carbonate ions at equilibrium. [↑](#footnote-ref-2)
2. For these two experiments, otolith growth data were measured by the growth increments along the distal axis from the starting date of the experiments. [↑](#footnote-ref-3)
3. Translucent otolith zones are structures that appear as a dark zone under reflected light (body text, Fig. 1, left panel).

   [↑](#footnote-ref-4)
4. The temperature conditions for the Norwegian coastal cod stock were derived from monthly temperatures at a 50 m depth averaged over the 1968-1993 period at Stasjoner. Data were taken from [http://www.imr.no/forskning/forskningsdata/stasjoner](http://www.imr.no/forskning/forskningsdata/stasjoner/dato.php?page=0&year=2009&stid=5863). [↑](#footnote-ref-5)
